# Minimally invasive aortic valve replacement through a right anterior minithoracotomy 306

**DOI:** 10.1186/1749-8090-10-S1-A53

**Published:** 2015-12-16

**Authors:** Durgaprasad Reddy Baluredy, Vikas Singarajipura Ramakrishna, Mayuri Stristava, Subhash Kumar Kadim

**Affiliations:** 1 Department of Cardiothoracic and Vascular Surgery, Vydehi Institute of Medical Sciences and Research Centre, Bangalore, Karnartaka, 560066, India

## Background/Introduction

The Aortic valve has been traditionally approached through a median sternotomy. However, significant advances in technology have allowed for Aortic valve surgery to be performed using progressively smaller incisions including the minithoracotomy and hemisternotomy.

## Aims/Objectives

To review the results of minimally invasive aortic valve replacement (AVR) through a right anterior minithoracotomy.

## Method

From July 2011 to January 2014, a total of 84 patients with isolated aortic valve disease (rheumatic in 62 patients, degenerative in 14 patients, congenital in 8 patients) underwent AVR through a right anterior minithoracotomy approach in the third intercostal space with a groin incision for femoral cannulation.

## Results

The mean age was 41.4 years (ranging from 19 to 74 years). 58 patients were male. Mean duration of cardiopulmonary bypass time and aortic cross-clamp time was (82 ± 23) minutes and (53 ± 16) minutes, respectively. 2 patients required conversion to median sternotomy. The mean hospital stay was 4 ± 1 day. Follow-up was performed in all patients for a period of 15 ± 6 months postoperatively. A good recovery was obtained in all patients.

## Discussion/Conclusion

Minimally invasive aortic valve replacement though the right anterior minithoracotomy approach is safe and feasible with reduced postoperative recovery time.

